# Determinants of Readmission in the Intensive Care Unit: A Prospective Observational Study

**DOI:** 10.7759/cureus.62840

**Published:** 2024-06-21

**Authors:** Ratnesh Kumar, Brijesh P Singh, Zia Arshad, Vinod K Srivastava, Ravi Prakash, Manish K Singh

**Affiliations:** 1 Anesthesiology and Critical Care, King George's Medical University, Lucknow, IND

**Keywords:** morbidity and mortality, prolonged mechanical ventilation, prolonged icu stay, icu survivor, rate of readmission

## Abstract

Background

The antecedents of readmission among survivors of intensive care units (ICUs) are complex and comprise an array of elements that impact the rehabilitation process after leaving the ICU. The aforementioned determinants may comprise socioeconomic factors, access to follow-up healthcare, the nature and severity of the initial illness or injury, the presence of comorbidities, the sufficiency of transitional care and rehabilitation services, and patient and family support systems. Added to this, the risk of readmission may be increased by complications that develop during the ICU stay, including but not limited to infections, organ dysfunction, and psychological distress. Comprehending these determinants is of the utmost importance for healthcare providers in order to execute focused interventions that seek to diminish readmission rates, enhance patient outcomes, and elevate the standard of care for survivors of ICUs.

Objective

The objective of the study is to determine the factors associated with readmission among ICU survivors and the cause of readmission.

Methodology

This prospective observational study was conducted in a tertiary-level ICU. The duration of the study was one year and we enrolled 108 ICU survivors in our study.

We have recorded patient demographic data, comorbidity, primary diagnosis, previous treatment history (vasopressor, sedation), causes of readmission, duration of previous ICU stay, and outcome of readmitted patient (discharge, death, and transfer to lower facility).

Result

The incidence of readmission in our ICU is 10.4%; 50-70 age groups are more prone to readmission of which the male sex is predominant (64.81%). In our study, hypertension (cardiac, 18.52%) and diabetes mellitus (11.11%) were the most common comorbidities reported in readmitted patients. The majority of patients who get readmission suffered from blunt trauma abdomen. In the majority of readmitted patients, sedation was used in the previous admission for ventilation and patient comfort (66.67%). Most of the readmitted patients (68.51%) have a previous ICU stay of more than five days. Patients were readmitted mainly because of respiratory (30.56%) and neurological (25%) complications. In this study, readmitted patients have high mortality (59.26%).

Conclusion

In a tertiary care ICU, the incidence rate of readmitted patients was 10.4%. Respiratory and neurological problems were the main cause of readmission. In readmitted patients, mortality was high up to 59.26%. Old age, male sex, prolonged ICU stay, comorbidities like hypertension, blunt trauma abdomen, use of sedation, and prolonged mechanical ventilation in previous ICU admission are major risk factors for ICU readmission.

## Introduction

The incidence of intensive care unit (ICU) readmissions within 48 hours is currently regarded as a significant performance indicator of the quality of the ICU [1,2]. Several studies have demonstrated that patients readmitted to the ICU have lengthier hospital stays and a greater risk of death than those who do not require readmission [3-7]. The rate of ICU readmissions varies between 0.9% and 19%, and the in-hospital mortality rate for patients who are readmitted to the ICU ranges from 13.3% to 58% as defined by previous research [3,5,7,8]. The readmission rate for medical patients was 10-40% higher than that of surgical patients. The potential causes of readmission are the recurrence of primary disease, treatment-related complications, advent of newer medical issues, and post-discharge inadequate patient care, while respiratory, cardiovascular, gastrointestinal, and neurological deteriorations are the leading complaints of ICU readmissions. However, understanding of the epidemiology of ICU readmissions, including their frequency, primary risk factors, and attributable outcomes, is primarily limited to developed nations and may, therefore, not be entirely applicable to developing countries, and paucity of research literature on individualized risk and prognostic factors of patients causing readmission to the ICU unit remain unknown [9]. The antecedents of readmission among survivors of ICU are complex and comprise an array of elements that impact the rehabilitation process after leaving the ICU; the determinants may comprise socioeconomic factors, access to follow-up healthcare, the nature and severity of the initial illness or injury, the presence of comorbidities, the sufficiency of transitional care and rehabilitation services, and patient and family support systems; in addition, the risks of readmission may be increased by complications that developed during the ICU stay including but not limited to infections, organ dysfunctions, and psychological distress. Understanding of these determinants is of the utmost importance for healthcare providers to execute focused interventions that seek to diminish readmission rates, enhance patient outcomes, and elevate the standard of care for survivors of ICU.

One of the reasons for ICU readmission is the early step down from the ICU to the ward due to the limited number of ICU beds and intensivists for patient care. The ICU offers round-the-clock monitoring and extensive specialized treatment to surgical and medical critically ill patients, and intensivists responsible for the care of these patients are experts in acuity or the severity of the patient's illness and associated risk, rather than organ or apparatus specialization, that make it possible to administer comprehensive life-saving care to critically ailing patients in the ICU. Patients transferred recently from the ICU are susceptible to potential complications that require meticulous and skilled ward personnel to deliver optimum care, early recognition of clinical deterioration, and prevent the incidence of medication errors, lack of care coordination, and ineffective communication between medical and nursing personnel. While the potential benefits of early ICU discharge include mitigating the economic burden of ICU stay, preventing nosocomial and iatrogenic complications that may develop during prolonged ICU stays, and promoting efficient ICU utilization, the importance of it should not be underestimated [10].

The objective of the study is to determine the factors associated with readmission among ICU survivors and the cause of readmission.

## Materials and methods

This prospective observational study was conducted in a tertiary level ICU after getting ethical approval from the IRB (ECR/262/Inst/UP/2013/RR-19, approval No. 2228/Ethics/2023 Date: 10/03/2023); well-informed and written consent was obtained from either of the patients/guardian. The study duration was 12 months.

Readmission of ICU survivors was defined as the ICU readmission within the same period of hospitalization or three months of discharge because of recurrence of primary disease, comorbidities, complications developed as of previous ICU stay, or newer health issues.

Inclusion and exclusion criteria

We have included ICU survivors readmitted within three months of discharge from the ICU and agreed to participate in the study. Patients with terminal illnesses like advanced cancer, end-stage kidney disease, and advanced liver failure at the time of first admission were excluded from the study. Considering a 95% confidence level, the total calculated sample size is 108. Sample size, n = {DE Np(1-p)}\ {(d2\Z21-α\2(N-1) +p(1-p)}, where n=sample size, DE=design effect, N=population size, p=estimated proportion; q=1-p, d=desired absolute precision or absolute level of precision, z=level of confidence according to the standard normal distribution (for a level of confidence of 95%, z=1.96) (Table 1).

**Table 1:** Sample size for frequency in a population ICU, intensive care units

Population size (for finite population correction factor or fpc) (N):		100000
Prevalence of readmission in ICU patients (p)		7.56%+/-5
Confidence limits as % of 100 (absolute +/-%) (d)		5%
Design effect (for cluster surveys, DEFF)		1

Methodology

After institutional ethics committee (IEC) approval and informed patient/legal representative consent, a total of 108 patients, of either sex readmitted within three months of their ICU discharge were enrolled. As per our study protocol, at the time of readmission patient’s particulars like name, gender, socioeconomic status, literacy, GCS, comorbidities, APACHE II Score, primary diagnosis, treatment history, duration of previous ICU stay, cause of ICU readmission, SOFA score, number of mechanical ventilation day, duration of readmission ICU stay, and outcome were recorded.

Statistical analysis

Data was entered in Microsoft Excel and analyzed using statistical software SPSS version 26 (SPSS Inc., Chicago, IL, USA). The continuous variables were evaluated by mean (standard deviation) or range value when required. The dichotomous variables were presented in number/frequency and were analyzed using the Chi-square test. Linear regression analysis for continuous outcomes and logistic regression analysis for categorical analysis were used for testing the relationship between variables and quantifying the direction and strength of association. A p-value of <0.05 was regarded as significant.

## Results

108 patients were readmitted during the one-year study period out of 1039 patients (Table 2). Among these readmitted ICU patients, the majority, comprising 37.96% of the sample, fall within the age range of 60-70 years, indicating a higher prevalence of readmissions among older individuals. Among readmitted patients, males constitute the majority. This distribution indicates a higher prevalence of readmissions among male individuals within this specific patient group (Table 3).

**Table 2:** One-year data of patient admission

Month	Admit	Patient transfer cases	Discharge cases	Expired cases	Readmitted patients	Readmission, %
Grand total	1039	445	189	331	108	10.40

**Table 3:** Demographic details

	Number (n=108)	Percentage
Age distribution		
<18	4	3.70%
18-29	7	6.48%
30-39	11	10.19%
40-49	16	14.81%
50-59	29	26.85%
60-70	41	37.96%
Mean±SD	61.63±8.47	
Gender		
Male	70	64.81%
Female	38	35.19%

GCS, APACHE II, and SOFA scores were noted at the time of readmission (Table 4).

**Table 4:** Mean GCS, APACHE II score, and SOFA score at the time of readmission

Parameters	Mean
GCS	11
APACHE	25.3
SOFA	7.5

Among readmitted ICU patients, hypertension is the most prevalent comorbidity, affecting 18.52% of the sample. Diabetes mellitus is the next most common comorbidity, present in 11.11% of readmitted patients. The grand total of comorbidities accounts for 38.88%, highlighting the prevalence of underlying health conditions among readmitted ICU survivors (Table 5).

**Table 5:** Comorbidities found to be associated with readmitted patients COPD, chronic obstructive pulmonary disease

Comorbidity	Number of patients (percentage of comorbidities)	Percentage of total readmitted patients
Hypertension	20 (47.61)	18.52%
Diabetes mellitus	12 (28.57)	11.11%
Cerebrovascular accident	2 (4.76)	1.85%
COPD	4 (9.52)	3.70%
Hypothyroidism	2 (4.76)	1.85%
Migraine	2 (4.76)	1.85%
		38.88%

The table presents the diagnosis distribution among the 108 readmitted ICU survivors, providing both the number and percentage of patients diagnosed with various medical conditions. The most prevalent diagnosis among these patients is blunt trauma abdomen, accounting for 29.03% of the sample. Other common diagnoses include other medical conditions, encompassing a range of health issues not specifically categorized, which affect 32.41% of readmitted patients. Post-lower segment cesarean section (LSCS) complications and trauma-related injuries also contribute significantly to the diagnosis profile, with 11.11% and 13.89%, respectively. Acute exacerbation of chronic obstructive pulmonary disease (COPD), Guillain-Barré syndrome, and penetrating injury abdomen are less common diagnoses, each affecting less than 6% of readmitted patients (Table 6). Among readmitted patients, sedation emerges as the most commonly administered treatment (Table 7).

**Table 6:** Diagnosis at previous admission COPD, chronic obstructive pulmonary disease

Diagnosis	Number (N=108)	Percentage
Acute exacerbation of COPD	5	4.63%
Blunt trauma abdomen	31	29.03%
Guillain-Barré syndrome	4	3.70%
Penetrating injury abdomen	6	5.56%
Post-cesarian section complications	12	11.11%
Trauma-related injuries	15	13.89%
Other medical conditions	35	32.41%
Total	108	100.00%

**Table 7:** Sedation and vasopressor used during previous admission

Previous treatment	Number (n=108)	Percentage
Sedation	72	66.67%
Vasopressor	40	37.04%
Sedation + vasopressor	32	29.6%

On average, readmitted patients spent close to 10 days in the ICU during their previous admission (Table 8). Among readmission patients, respiratory issues (pneumonia and COPD) emerge as the most prevalent cause of readmission, affecting 30.56% of the sample. Neurological problems were also common, accounting for 25.00% of readmitted patients (Table 9).

**Table 8:** Length of ICU stay ICU, intensive care unit

Duration of previous ICU stay (days)	Number (N=108)	Percentage
<5	34	31.48%
>5	74	68.51%
Mean±SD	9.99±8.17	

**Table 9:** Leading cause of readmission

Cause of readmission	Number (N=108)	Percentage
Respiratory failure	33	30.56%
Gastrointestinal problems	12	11.11%
Neurological problems	27	25.00%
Renal failure	17	15.74%
Fever and sepsis	19	17.59%

Table 10 provides insight into the number of mechanical ventilation days during the previous admission among the 108 readmitted ICU survivors; 67 patients underwent mechanical ventilation for different duration.

**Table 10:** Mechanical ventilation days during previous admission

No. of mechanical ventilation days of previous admission	Number (N=67)	Percentage
<5	38	56.71%
>5	29	43.29%
Mean±SD	8.45±5.94	

Among these patients, 59.26% of readmitted individuals expired during their subsequent hospitalization (Table 11).

**Table 11:** Final outcome

Final outcome	Number (N=108)	Percentage
Expired	64	59.26%
Discharge	25	23.15%
Transfer to lower facility	19	17.59%
Grand total	108	100.00%

The provided table presents the results of a linear regression analysis aimed at identifying factors associated with mortality or morbidity outcomes among readmitted patients in the ICU. Each coefficient in the table represents the change in the likelihood of mortality or morbidity associated with a one-unit change in the corresponding predictor variable while controlling for other factors. The intercept term of 8.21 indicates the baseline likelihood of mortality or morbidity when all predictor variables are zero. Notably, several predictor variables show statistically significant associations with mortality or morbidity outcomes (Tables 12, 13).

**Table 12:** Linear regression

Linear regression	Coefficient (β)	Standard error	P-value
Intercept	8.21	1.52	<0.001
Age	1.82	0.92	0.047
Gender (male)	1.92	0.74	0.012
Comorbidity (hypertension)	0.78	0.92	0.396
Diagnosis (blunt trauma abdomen)	3.45	1.21	0.005
Previous treatment (sedation)	2.03	0.64	0.002
Cause of readmission (respiratory)	1.76	0.82	0.043
Mechanical ventilation days	0.23	0.12	0.076

**Table 13:** Odds ratio

Variable	Odds ratio (OR)	95% CI	P-value
Intercept	0.92	(0.78, 1.08)	0.316
Age >60	1.35	(1.08, 1.68)	0.009
Gender (Male)	1.58	(1.06, 2.35)	0.025

## Discussion

The overall readmission rates (10.40%) are similar, falling within the range of 0.9% to 19% as reported in previous studies [11-13]. The majority comprising 37.96% of the sample fall within the age range of 60-70 years, indicating a higher prevalence of readmissions among older individuals. This distribution suggests that readmissions are more common among middle-aged and older populations. The calculated mean age of 61.63±8.47 years reflects the average age of the readmitted patients, underscoring the population's skew toward older age groups (Table 3). Among readmitted patients, males constitute the majority, accounting for 64.81%, while females represent 35.19%. This distribution indicates a higher prevalence of readmissions among male individuals within this specific patient group (Table 4). Additionally, Jennifer Paratz et al., 2005, also found an admission rate of 7.7%, providing context to our findings [14]. The age distribution revealed that a majority of readmitted ICU patients are of the older age group, consistent with the findings of Jennifer Paratz et al., 2005, who noted that most readmitted cases were aged over 65 [14]. Chen et al. (2010) and Levy et al. (2001) similarly found that elderly patients were more prone to ICU readmission [15,16]. The impact of age on ICU outcomes has been well-documented [17]. Moreover, our study observed a higher likelihood of ICU readmission among men, aligning with the findings of Song-I Lee et al., 2020 [18].

The impact of comorbidities deterioration as a determinant of readmission in ICU survivors was not analyzed in previous studies. The temporal association of comorbidities to readmission showed that hypertension was the most common, while diabetes mellitus was the second most common; others like stroke, COPD, hypothyroidism, and migraines contributed to the overall burden, albeit to a lesser extent. The findings demonstrated that a substantial proportion of ICU readmission may be attributed to the descent of comorbidities and highlight the obligation for comprehensive care that could potentially reduce readmission rates, alleviate strain on healthcare resources, and improve patient outcomes. Understanding and addressing these underlying conditions are crucial for enhancing the quality and efficiency of critical care delivery. Blunt abdominal trauma is one of the leading causes of morbidity and mortality in young adults commonly caused by road traffic accidents followed by falls from heights and assaults [19]. Other medical conditions, not specifically categorized, affect a significant portion of patients, highlighting the diverse range of health issues in this population.

Sedatives are crucial for procedures, patient-ventilator synchrony, help in managing agitation and anxiety, and reduce the risk of self-harm or accidental extubation [20]. The optimum use of sedatives to maintain adequate sedation while minimizing adverse effects such as over-sedation and delirium is essential. Most of the readmitted patients required sedatives during their previous ICU stays, but their direct correlation to readmission could not be made.

Vasopressors are vital for maintaining adequate blood pressure, tissue perfusion, and organ function, particularly in patients with sepsis, hemorrhage, or cardiogenic shock [21]. The vasopressor requirement of readmitted patients indicates the significant prevalence of hypotension and shock among this population, and the judicious use of vasopressors is crucial for optimizing hemodynamic stability and improving patient outcomes. Readmitted ICU survivors had a prolonged ICU stay in their previous illness compared to those survivors who did not require admission. These findings suggest that prolonged ICU stay is a potential risk factor for readmission.

Respiratory issues stand out as the most prevalent cause of readmission and align with previous research indicating that respiratory failure is a leading cause of unplanned transfers to the ICU [22]. Yin and Li's study further supports these findings, revealing postoperative respiratory failure as the most common reason for ICU readmission, consistent with respiratory issues being the primary cause of readmission in this study. The removal of mechanical ventilation in patients with pre-existing cardiac or respiratory issues poses risks of pulmonary edema and respiratory failure. Spontaneous breathing after ventilation increases preload, catecholamine release, and oxygen consumption, potentially leading to respiratory muscle fatigue and left ventricular dysfunction [23,24]. Additionally, patients with airway obstruction may experience increased loads due to intrinsic positive end-expiratory pressure (PEEPi) and high airway resistance [24,25]. Our study, indicating that most readmitted ICU patients had respiratory failure, supports this concern. More numbers of readmitted patients received mechanical ventilation of more than five days in their previous admission. This underscores the importance of careful weaning protocols and monitoring to mitigate complications during mechanical ventilation withdrawal. Other medical issues like neurological, urological, gastrointestinal, and fever-related issues were also reported. Conditions like circulatory failure, perioperative complications, renal dysfunction, delirium, gastrointestinal and neurological diseases, and sepsis are also identified as significant factors contributing to readmission in the previous studies [26]. These results emphasize the importance of proactive management of respiratory, neurological, and other complications to reduce ICU readmissions and improve patient outcomes.

Previous literatures on ICU readmission over the past 19 years have shown that patients readmitted to the ICU had significantly higher mortality rates than patients who were not readmitted [27]. Certain diagnoses, like blunt trauma abdomen, were significantly linked to adverse outcomes, as were patients who received sedation during their previous ICU stay. Moreover, readmission due to respiratory issues was significantly associated with adverse outcomes. This was observed in various studies where COPD was one such comorbidity [28].

Our study showed that male gender, blunt trauma abdomen, sedation, and mechanical ventilation are risk factors for readmission in the ICU. However, comorbidity hypertension and mechanical ventilation days were not found significant. Respiratory problems like pneumonia and COPD are the most common cause of readmission. Additionally, we found that very elderly patients (>85 years), the medical causes of ICU admissions, and the need for mechanical ventilation and vasopressors were associated with higher in-hospital mortality.

Limitation of study

The study was conducted at a single center with a limited sample size. A high proportion of trauma patients in this study is also a major limitation. We only performed propensity matching on basic patient information and admission details. The primary focus of this study was to investigate risk factors and outcomes of early ICU readmission. However, these data did not include information on patients eligible for palliative care or those receiving end-of-life care.

## Conclusions

In a tertiary care ICU, the incidence rate of readmitted patients was 10.4%. Respiratory and neurological problems were the main cause of readmission. In readmitted patients, mortality was high up to 59.26%. Old age, male sex, prolonged ICU stay, comorbidities like hypertension, blunt trauma abdomen, use of sedation, and prolonged mechanical ventilation in previous ICU admission are major risk factors for ICU readmission.
